# Ethyl (4-{[(di­ethyl­carbamo­thio­yl)sulfan­yl]meth­yl}-2-oxo-2*H*-chromen-7-yl)carbamate

**DOI:** 10.1107/S1600536814001706

**Published:** 2014-01-29

**Authors:** K. R. Roopashree, K. Mahesh Kumar, B. R. Anitha, A. J. Ravi, H. C. Devarajegowda

**Affiliations:** a Department of Physics, Yuvaraja’s College (Constituent College), University of Mysore, Mysore 570 005, Karnataka, India; b Department of Chemistry, Karnatak University’s Karnatak Science College, Dharwad, Karnataka 580 001, India

## Abstract

In the title compound, C_18_H_22_N_2_O_4_S_2_, the 2*H*-chromene ring system is essentially planar (r.m.s. deviation = 0.012 Å). The mol­ecular conformation is stabilized by a C—H⋯O hydrogen bond. In the crystal, N—H⋯S and C—H⋯O hydrogen bonds occur, the former enclosing an *R* ^2^_2_(22) ring motif, and lead to the formation of a two-dimensional slab-like network lying parallel to (10-1). π–π inter­actions are observed between inversion-related aromatic rings [shortest centroid–centroid distance = 3.6300 (11) Å].

## Related literature   

For biological applications of coumarins and di­thio­carbamates, see: Cao *et al.* (2005 ▶); Chen *et al.* (2008 ▶); Gerhauser *et al.* (1997 ▶); Mehta *et al.* (1995 ▶); Valizadeha & Shockravi (2005 ▶); Zhang *et al.* (2005 ▶). For a related structure with comparable bond lengths and for the synthesis, see: Kumar *et al.* (2012 ▶).
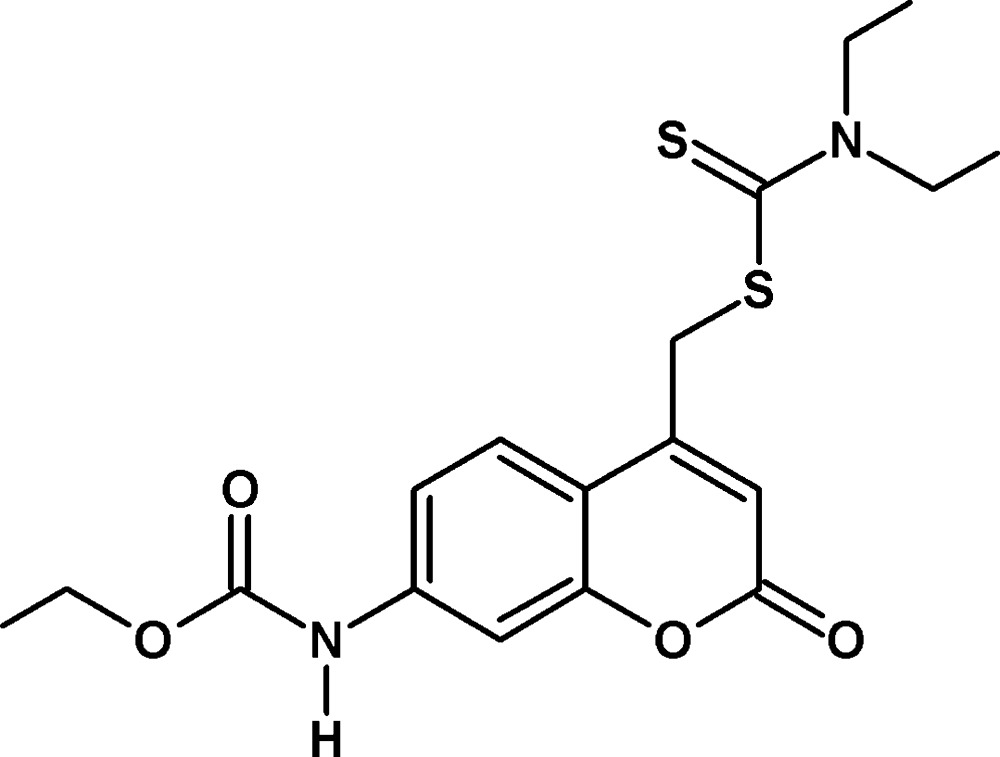


## Experimental   

### 

#### Crystal data  

C_18_H_22_N_2_O_4_S_2_
*M* *_r_* = 394.50
Triclinic, 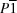

*a* = 8.0573 (3) Å
*b* = 9.0358 (4) Å
*c* = 14.2400 (6) Å
α = 74.137 (3)°
β = 87.831 (3)°
γ = 73.320 (2)°
*V* = 954.36 (7) Å^3^
*Z* = 2
Mo *K*α radiation
μ = 0.31 mm^−1^
*T* = 296 K
0.24 × 0.20 × 0.12 mm

#### Data collection  

Bruker SMART CCD area-detector diffractometer
Absorption correction: multi-scan (*SADABS*; Sheldrick, 2007 ▶) *T*_min_ = 0.770, *T*_max_ = 1.000
14877 measured reflections
3305 independent reflections
2808 reflections with *I* > 2σ(*I*)
*R*_int_ = 0.031

#### Refinement  

*R*[*F* ^2^ > 2σ(*F* ^2^)] = 0.035
*wR*(*F* ^2^) = 0.101
*S* = 0.95
3305 reflections
235 parameters
H-atom parameters constrained
Δρ_max_ = 0.25 e Å^−3^
Δρ_min_ = −0.17 e Å^−3^

### 

Data collection: *SMART* (Bruker, 2001 ▶); cell refinement: *SAINT* (Bruker, 2001 ▶); data reduction: *SAINT*; program(s) used to solve structure: *SHELXS97* (Sheldrick, 2008 ▶); program(s) used to refine structure: *SHELXL97* (Sheldrick, 2008 ▶); molecular graphics: *ORTEP-3 for Windows* (Farrugia, 2012 ▶); software used to prepare material for publication: *SHELXL97*.

## Supplementary Material

Crystal structure: contains datablock(s) I, global. DOI: 10.1107/S1600536814001706/bt6958sup1.cif

Structure factors: contains datablock(s) I. DOI: 10.1107/S1600536814001706/bt6958Isup2.hkl

Supporting information file. DOI: 10.1107/S1600536814001706/bt6958Isup3.cml

CCDC reference:

Additional supporting information:  crystallographic information; 3D view; checkCIF report

## Figures and Tables

**Table 1:** Hydrogen-bond geometry (Å, °)

*D*—H⋯*A*	*D*—H	H⋯*A*	*D*⋯*A*	*D*—H⋯*A*
N7—H7⋯S2^i^	0.86	2.63	3.4858 (17)	172
C17—H17⋯O4	0.93	2.28	2.876 (2)	121
C25—H25*B*⋯O6^ii^	0.97	2.49	3.306 (3)	142

Symmetry codes: (i) 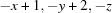; (ii) 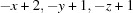.

**Fig. 1.**
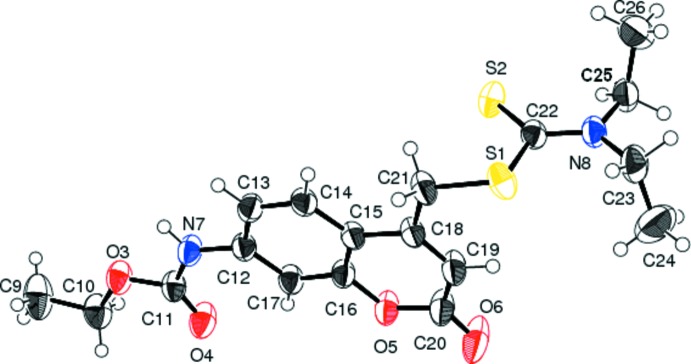
The molecular structure of the title compound. Displacement ellipsoids are drawn at the 50% probability level. Hydrogen atoms are shown as spheres of arbitrary radius.

**Fig. 2.**
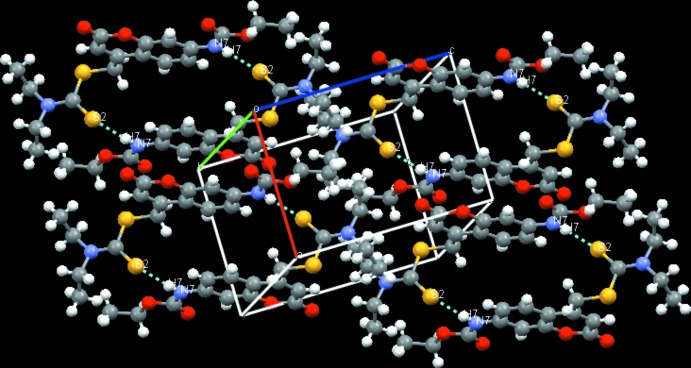
The crystal packing of the title compound. N-H···S hydrogen bonds are drawn as dashed lines

## supplementary crystallographic information

### 1. Comment

Coumarin and its derivatives are an important class of heterocyclic compound and a number of preparations have been known since late 19th century. Various compounds possessing an azomethine linkage at C-7 have been synthesized and evaluated for their anti-oxidant ability and anti-inflammatory activity. They showed 58% and 54% inhibition of inflammation induced by Carrageenan, which was better than the standard indomethacin (Valizadeha *et al.*, 2005). The luminescent property of Europium chelates of acylated 7-amino coumarins has been used to label proteins at cysteine residues on synthetic oligonucleotides containing a free thiol group (Chen *et al.*, 2008).

Organic dithiocarbamates have attracted a great deal of interest due to their interesting chemistry and wide utility (Zhang *et al.*, 2005). Dithiocarbamates have a wide range of uses and applications and are produced in great quantities throughout the world. Since, brassinin (Mehta *et al.*, 1995) a crucial plant defense first isolated from cabbage, had cancer preventive activity, structural modification on this compound led to the synthesis of sulforamate (Gerhauser *et al.*, 1997) and a series of dithiocarbamates, some of which were found to have in-vitro and in-vivo antitumor activity (Cao *et al.*, 2005). A steadily increasing number of structural studies have been published from our research group on dithiocarbamates. Based on the above literature survey we synthesized the title molecule for its chemical and physical studies.

The asymmetric unit of Ethyl (4-{[(diethylcarbamothioyl)thio]methyl} -2-oxo-2*H*-chromen-7-yl)carbamate is shown in Fig. 1. The 2*H* -chromene ring system is nearly planar, with a maximum deviation of 0.0149 (18) Å for the atom C13. The molecular conformation is stabilized by a C-H···O hydrogen bond and the crystal packing is stabilized by N—H···S and C–H···O hydrogen bonds (Table 1). The N—H···S hydrogen bonds form an *R*_2_^2^(22) ring pattern. In addition, π–π interactions between inversion related molecules are also observed: Cg1···Cg2^iii^ 3.6300 (11) Å [Cg1 is the centroid of the ring formed by C12 to C17, Cg2 is the centroid of the ring formed by O5,C15,C16,C18,C19,C20; symmetry operator (iii): -*x*+2, -*y*+2, -*z*] (Fig. 2).

### 2. Experimental

All the chemicals used were of analytical reagent grade and were used directly without further purification. The title compound was synthesized according to the reported method7 (Kumar *et al.*, 2012). The compound is recrystallized by ethanol-chloroform mixture. Colourless needles of the title compound were grown from a mixed solution of Ethanol/Chloroform (V/V = 2/1) by slow evaporation at room temperature. Yield= 91%, m.p. 445 K.

### 3. Refinement

All H atoms were positioned geometrically, with N—H = 0.86 Å, C—H = 0.93 Å for aromatic H, C—H = 0.97 Å for methylene H and C—H = 0.96 Å for methyl H and were refined using a riding model with *U*_iso_(H) = 1.5*U*_eq_(C) for methyl H and *U*_iso_(H) = 1.2*U*_eq_(C,N) for all other H.

### Crystal data

**Table:**

C_18_H_22_N_2_O_4_S_2_	*Z* = 2
*M**_r_* = 394.50	*F*(000) = 416
Triclinic, *P*1	*D*_x_ = 1.373 Mg m^−^^3^
Hall symbol: -P 1	Melting point: 445 K
*a* = 8.0573 (3) Å	Mo *K*α radiation, λ = 0.71073 Å
*b* = 9.0358 (4) Å	Cell parameters from 3305 reflections
*c* = 14.2400 (6) Å	θ = 1.5–25.0°
α = 74.137 (3)°	µ = 0.31 mm^−^^1^
β = 87.831 (3)°	*T* = 296 K
γ = 73.320 (2)°	Plate, colourless
*V* = 954.36 (7) Å^3^	0.24 × 0.20 × 0.12 mm

### Data collection

**Table:**

Bruker SMART CCD area-detector diffractometer	3305 independent reflections
Radiation source: fine-focus sealed tube	2808 reflections with *I* > 2σ(*I*)
Graphite monochromator	*R*_int_ = 0.031
ω and φ scans	θ_max_ = 25.0°, θ_min_ = 1.5°
Absorption correction: multi-scan (*SADABS*; Sheldrick, 2007)	*h* = −9→9
*T*_min_ = 0.770, *T*_max_ = 1.000	*k* = −10→10
14877 measured reflections	*l* = −16→16

### Refinement

**Table:**

Refinement on *F*^2^	Primary atom site location: structure-invariant direct methods
Least-squares matrix: full	Secondary atom site location: difference Fourier map
*R*[*F*^2^ > 2σ(*F*^2^)] = 0.035	Hydrogen site location: inferred from neighbouring sites
*wR*(*F*^2^) = 0.101	H-atom parameters constrained
*S* = 0.95	*w* = 1/[σ^2^(*F*_o_^2^) + (0.0672*P*)^2^ + 0.1652*P*] where *P* = (*F*_o_^2^ + 2*F*_c_^2^)/3
3305 reflections	(Δ/σ)_max_ < 0.001
235 parameters	Δρ_max_ = 0.25 e Å^−^^3^
0 restraints	Δρ_min_ = −0.17 e Å^−^^3^

### Special details

**Table:**

Experimental. IR (KBr, cm-1): 1000, 1152, 1227, 1422, 1501, 1695, 1720, 3265. GCMS: m/e: 384. 1H NMR (400 MHz, CDCl_3_, δ,. p.p.m): 7.68 (dd, 1H, Ar—H), 7.36 (d, 1H, Ar—H), 7.26 (s, 1H, Ar—H), 6.91 (s, 1H, NH), 6.46 (s, 2H, Ar—H), 4.71 (s, 2H, CH_2_), 4.29 (q, 2H, CH_2_), 4.06 (q, 2H, CH_2_), 3.78 (q, 2H, CH_2_), 1.59 (s, 3H, CH_3_), 1.35 (m, 6H, CH_3_). Mol. Formula: C_18_H_22_N_2_O_4_S_2_. Elemental analysis: C, 54.80; H, 5.62; N, 7.10 (calculated); C, 54.84; H, 5.58; N, 7.14 (found).
Geometry. All s.u.'s (except the s.u. in the dihedral angle between two l.s. planes) are estimated using the full covariance matrix. The cell s.u.'s are taken into account individually in the estimation of s.u.'s in distances, angles and torsion angles; correlations between s.u.'s in cell parameters are only used when they are defined by crystal symmetry. An approximate (isotropic) treatment of cell s.u.'s is used for estimating s.u.'s involving l.s. planes.
Refinement. Refinement of *F*^2^ against ALL reflections. The weighted *R*-factor *wR* and goodness of fit *S* are based on *F*^2^, conventional *R*-factors *R* are based on *F*, with *F* set to zero for negative *F*^2^. The threshold expression of *F*^2^ > 2σ(*F*^2^) is used only for calculating *R*-factors(gt) *etc*. and is not relevant to the choice of reflections for refinement. *R*-factors based on *F*^2^ are statistically about twice as large as those based on *F*, and *R*- factors based on ALL data will be even larger.

### Fractional atomic coordinates and isotropic or equivalent isotropic displacement parameters (Å^2^)

**Table:**

	*x*	*y*	*z*	*U*_iso_*/*U*_eq_	
S1	0.95674 (6)	0.46925 (5)	0.22606 (3)	0.04992 (16)	
S2	0.60616 (6)	0.62085 (5)	0.29583 (3)	0.05289 (16)	
O3	0.57157 (17)	1.67152 (13)	−0.18754 (9)	0.0517 (3)	
O5	0.95552 (18)	1.08031 (13)	0.18541 (8)	0.0512 (3)	
O4	0.7317 (2)	1.58432 (15)	−0.04675 (10)	0.0616 (4)	
O6	1.1139 (3)	0.93665 (17)	0.31862 (11)	0.0900 (6)	
N7	0.6313 (2)	1.41614 (16)	−0.10823 (10)	0.0480 (4)	
H7	0.5682	1.4173	−0.1559	0.058*	
N8	0.84521 (18)	0.36613 (16)	0.39956 (10)	0.0431 (3)	
C9	0.4882 (4)	1.9375 (2)	−0.28981 (17)	0.0794 (7)	
H9A	0.4919	2.0456	−0.2983	0.119*	
H9B	0.3697	1.9354	−0.2885	0.119*	
H9C	0.5431	1.8991	−0.3431	0.119*	
C10	0.5810 (3)	1.8330 (2)	−0.19639 (14)	0.0562 (5)	
H10A	0.7010	1.8343	−0.1967	0.067*	
H10B	0.5267	1.8705	−0.1419	0.067*	
C11	0.6522 (2)	1.55978 (19)	−0.10780 (12)	0.0441 (4)	
C12	0.6996 (2)	1.26657 (19)	−0.04079 (11)	0.0404 (4)	
C13	0.6722 (2)	1.1326 (2)	−0.06140 (12)	0.0440 (4)	
H13	0.6091	1.1460	−0.1180	0.053*	
C14	0.7369 (2)	0.98170 (19)	0.00062 (11)	0.0413 (4)	
H14	0.7164	0.8944	−0.0143	0.050*	
C15	0.8334 (2)	0.95701 (18)	0.08610 (11)	0.0381 (4)	
C16	0.8600 (2)	1.09192 (19)	0.10417 (11)	0.0397 (4)	
C17	0.7951 (2)	1.24599 (19)	0.04376 (11)	0.0427 (4)	
H17	0.8147	1.3334	0.0591	0.051*	
C18	0.9075 (2)	0.80158 (19)	0.15570 (11)	0.0399 (4)	
C19	1.0006 (3)	0.7950 (2)	0.23349 (12)	0.0507 (5)	
H19	1.0483	0.6956	0.2780	0.061*	
C20	1.0302 (3)	0.9345 (2)	0.25129 (13)	0.0564 (5)	
C21	0.8756 (3)	0.65683 (19)	0.13488 (12)	0.0466 (4)	
H21A	0.9274	0.6457	0.0736	0.056*	
H21B	0.7515	0.6775	0.1257	0.056*	
C22	0.7977 (2)	0.47977 (18)	0.31595 (11)	0.0400 (4)	
C23	1.0135 (3)	0.2415 (2)	0.41870 (15)	0.0568 (5)	
H23A	1.0581	0.2235	0.3573	0.068*	
H23B	0.9972	0.1417	0.4592	0.068*	
C24	1.1436 (3)	0.2872 (4)	0.4689 (2)	0.0881 (8)	
H24A	1.2513	0.2030	0.4798	0.132*	
H24B	1.1011	0.3029	0.5304	0.132*	
H24C	1.1619	0.3849	0.4285	0.132*	
C25	0.7274 (2)	0.3530 (2)	0.48079 (12)	0.0513 (4)	
H25A	0.6537	0.4594	0.4797	0.062*	
H25B	0.7950	0.3090	0.5422	0.062*	
C26	0.6168 (3)	0.2487 (3)	0.47493 (17)	0.0731 (6)	
H26A	0.5419	0.2429	0.5291	0.110*	
H26B	0.6893	0.1428	0.4771	0.110*	
H26C	0.5480	0.2931	0.4148	0.110*	

### Atomic displacement parameters (Å^2^)

**Table:**

	*U*^11^	*U*^22^	*U*^33^	*U*^12^	*U*^13^	*U*^23^
S1	0.0656 (3)	0.0310 (2)	0.0483 (3)	−0.0110 (2)	0.0094 (2)	−0.00698 (18)
S2	0.0549 (3)	0.0449 (3)	0.0458 (3)	0.0002 (2)	−0.0096 (2)	−0.0051 (2)
O3	0.0703 (9)	0.0313 (6)	0.0473 (7)	−0.0103 (6)	−0.0114 (6)	−0.0034 (5)
O5	0.0787 (9)	0.0340 (6)	0.0398 (6)	−0.0181 (6)	−0.0181 (6)	−0.0036 (5)
O4	0.0918 (10)	0.0410 (7)	0.0514 (8)	−0.0213 (7)	−0.0214 (7)	−0.0062 (6)
O6	0.1509 (16)	0.0488 (8)	0.0661 (10)	−0.0303 (9)	−0.0594 (10)	0.0009 (7)
N7	0.0632 (10)	0.0345 (7)	0.0423 (8)	−0.0139 (7)	−0.0147 (7)	−0.0021 (6)
N8	0.0500 (8)	0.0322 (7)	0.0402 (7)	−0.0088 (6)	−0.0049 (6)	−0.0013 (6)
C9	0.127 (2)	0.0356 (10)	0.0623 (13)	−0.0109 (12)	−0.0103 (13)	−0.0040 (9)
C10	0.0799 (14)	0.0318 (9)	0.0540 (11)	−0.0136 (9)	−0.0023 (9)	−0.0088 (8)
C11	0.0536 (10)	0.0334 (9)	0.0388 (9)	−0.0076 (7)	−0.0026 (8)	−0.0040 (7)
C12	0.0464 (9)	0.0345 (8)	0.0364 (8)	−0.0117 (7)	−0.0014 (7)	−0.0031 (7)
C13	0.0530 (10)	0.0420 (9)	0.0367 (8)	−0.0169 (8)	−0.0083 (7)	−0.0054 (7)
C14	0.0535 (10)	0.0355 (8)	0.0382 (8)	−0.0192 (7)	−0.0010 (7)	−0.0086 (7)
C15	0.0473 (9)	0.0339 (8)	0.0323 (8)	−0.0138 (7)	0.0020 (7)	−0.0055 (6)
C16	0.0519 (10)	0.0357 (8)	0.0318 (8)	−0.0149 (7)	−0.0038 (7)	−0.0064 (6)
C17	0.0576 (11)	0.0322 (8)	0.0384 (9)	−0.0155 (7)	−0.0049 (7)	−0.0064 (7)
C18	0.0536 (10)	0.0336 (8)	0.0316 (8)	−0.0152 (7)	0.0029 (7)	−0.0045 (6)
C19	0.0743 (13)	0.0349 (9)	0.0381 (9)	−0.0152 (8)	−0.0093 (8)	−0.0011 (7)
C20	0.0847 (14)	0.0385 (9)	0.0422 (10)	−0.0184 (9)	−0.0198 (9)	−0.0014 (7)
C21	0.0703 (12)	0.0351 (9)	0.0348 (8)	−0.0203 (8)	0.0020 (8)	−0.0049 (7)
C22	0.0526 (10)	0.0294 (8)	0.0389 (8)	−0.0146 (7)	−0.0049 (7)	−0.0072 (6)
C23	0.0605 (12)	0.0352 (9)	0.0592 (11)	−0.0020 (8)	−0.0052 (9)	0.0009 (8)
C24	0.0551 (14)	0.0930 (18)	0.104 (2)	−0.0066 (13)	−0.0218 (13)	−0.0188 (15)
C25	0.0603 (12)	0.0476 (10)	0.0392 (9)	−0.0146 (9)	−0.0036 (8)	−0.0010 (7)
C26	0.0832 (16)	0.0737 (15)	0.0689 (14)	−0.0414 (13)	0.0086 (12)	−0.0107 (11)

### Geometric parameters (Å, º)

**Table:**

S1—C22	1.7781 (17)	C14—C15	1.400 (2)
S1—C21	1.7920 (16)	C14—H14	0.9300
S2—C22	1.6714 (17)	C15—C16	1.387 (2)
O3—C11	1.335 (2)	C15—C18	1.454 (2)
O3—C10	1.454 (2)	C16—C17	1.383 (2)
O5—C20	1.375 (2)	C17—H17	0.9300
O5—C16	1.377 (2)	C18—C19	1.340 (3)
O4—C11	1.202 (2)	C18—C21	1.509 (2)
O6—C20	1.201 (2)	C19—C20	1.436 (3)
N7—C11	1.357 (2)	C19—H19	0.9300
N7—C12	1.398 (2)	C21—H21A	0.9700
N7—H7	0.8600	C21—H21B	0.9700
N8—C22	1.326 (2)	C23—C24	1.498 (3)
N8—C25	1.470 (2)	C23—H23A	0.9700
N8—C23	1.471 (2)	C23—H23B	0.9700
C9—C10	1.484 (3)	C24—H24A	0.9600
C9—H9A	0.9600	C24—H24B	0.9600
C9—H9B	0.9600	C24—H24C	0.9600
C9—H9C	0.9600	C25—C26	1.490 (3)
C10—H10A	0.9700	C25—H25A	0.9700
C10—H10B	0.9700	C25—H25B	0.9700
C12—C17	1.394 (2)	C26—H26A	0.9600
C12—C13	1.398 (2)	C26—H26B	0.9600
C13—C14	1.371 (2)	C26—H26C	0.9600
C13—H13	0.9300		
			
C22—S1—C21	102.94 (8)	C19—C18—C15	118.47 (15)
C11—O3—C10	115.29 (14)	C19—C18—C21	124.04 (15)
C20—O5—C16	121.44 (13)	C15—C18—C21	117.48 (14)
C11—N7—C12	128.10 (15)	C18—C19—C20	122.95 (16)
C11—N7—H7	115.9	C18—C19—H19	118.5
C12—N7—H7	115.9	C20—C19—H19	118.5
C22—N8—C25	121.32 (14)	O6—C20—O5	116.39 (16)
C22—N8—C23	124.24 (15)	O6—C20—C19	126.28 (17)
C25—N8—C23	114.42 (14)	O5—C20—C19	117.33 (15)
C10—C9—H9A	109.5	C18—C21—S1	116.27 (12)
C10—C9—H9B	109.5	C18—C21—H21A	108.2
H9A—C9—H9B	109.5	S1—C21—H21A	108.2
C10—C9—H9C	109.5	C18—C21—H21B	108.2
H9A—C9—H9C	109.5	S1—C21—H21B	108.2
H9B—C9—H9C	109.5	H21A—C21—H21B	107.4
O3—C10—C9	107.06 (16)	N8—C22—S2	123.52 (13)
O3—C10—H10A	110.3	N8—C22—S1	113.94 (13)
C9—C10—H10A	110.3	S2—C22—S1	122.55 (9)
O3—C10—H10B	110.3	N8—C23—C24	112.18 (18)
C9—C10—H10B	110.3	N8—C23—H23A	109.2
H10A—C10—H10B	108.6	C24—C23—H23A	109.2
O4—C11—O3	124.67 (15)	N8—C23—H23B	109.2
O4—C11—N7	126.25 (15)	C24—C23—H23B	109.2
O3—C11—N7	109.07 (15)	H23A—C23—H23B	107.9
C17—C12—N7	123.04 (15)	C23—C24—H24A	109.5
C17—C12—C13	119.40 (15)	C23—C24—H24B	109.5
N7—C12—C13	117.55 (15)	H24A—C24—H24B	109.5
C14—C13—C12	120.94 (15)	C23—C24—H24C	109.5
C14—C13—H13	119.5	H24A—C24—H24C	109.5
C12—C13—H13	119.5	H24B—C24—H24C	109.5
C13—C14—C15	121.05 (15)	N8—C25—C26	111.89 (16)
C13—C14—H14	119.5	N8—C25—H25A	109.2
C15—C14—H14	119.5	C26—C25—H25A	109.2
C16—C15—C14	116.74 (14)	N8—C25—H25B	109.2
C16—C15—C18	118.47 (15)	C26—C25—H25B	109.2
C14—C15—C18	124.79 (15)	H25A—C25—H25B	107.9
O5—C16—C17	114.95 (14)	C25—C26—H26A	109.5
O5—C16—C15	121.31 (14)	C25—C26—H26B	109.5
C17—C16—C15	123.74 (15)	H26A—C26—H26B	109.5
C16—C17—C12	118.13 (15)	C25—C26—H26C	109.5
C16—C17—H17	120.9	H26A—C26—H26C	109.5
C12—C17—H17	120.9	H26B—C26—H26C	109.5

### Hydrogen-bond geometry (Å, º)

**Table:**

*D*—H···*A*	*D*—H	H···*A*	*D*···*A*	*D*—H···*A*
N7—H7···S2^i^	0.86	2.63	3.4858 (17)	172
C17—H17···O4	0.93	2.28	2.876 (2)	121
C25—H25*B*···O6^ii^	0.97	2.49	3.306 (3)	142

Symmetry codes: (i) −*x*+1, −*y*+2, −*z*; (ii) −*x*+2, −*y*+1, −*z*+1.
